# Matched T cell repertoire analysis of peripheral blood and tumor-infiltrating lymphocytes (TILs) in early stage breast cancer (ESBC) patients (pts) treated with pre-operative cryoablation (cryo) and/or Ipilimumab (Ipi)

**DOI:** 10.1186/2051-1426-2-S3-P138

**Published:** 2014-11-06

**Authors:** David B Page, Heather McArthur, Zhiwan Dong, Phillip Wong, Ryan Emerson, Zhenyu Mu, Chunjun Zhao, Christopher Comstock, Elizabeth Morris, Elizabeth Comen, Alan Kotin, Janice Sung, Edi Brogi, Monica Morrow, Stephen Solomon, Virgilio Sacchini, Majid Maybody, Deirdre Neville, Adi Diab, Padmanee Sharma, Harlan Robins, Sujata Patil, Jedd D Wolchok, Clifford Hudis, Larry Norton, James Allison, Jianda Yuan

**Affiliations:** 1 Memorial Sloan Kettering Cancer Center, New York, NY, USA; 2 Sloan Kettering Institute, New York, NY, USA; 3 Adative Biotechnologies, Seattle, WA, USA; 4 MD Anderson Cancer Center, Houston, TX, USA; 5 Fred Hutchinson Cancer Center, Seattle, WA, USA; 6 Immune Monitoring Facility, Sloan Kettering Institute, New York, NY, USA

## Background

Cryo plus anti-CTLA-4 therapy induces antigen-specific clonal T cell expansion, enhanced survival, and long-term anti-tumor immunity in mice [1]. We recently demonstrated that pre-operative cryo and/or anti-CTLA-4 therapy with Ipi is well tolerated and clinically feasible in women with ESBC. Furthermore, cryo with or without Ipi generates a polyclonal influx of novel T cell clones within the tumor bed [2,3]. Here, we utilize T cell repertoire analysis to explore the impact of cryo and/or Ipi on clonal expansion within peripheral blood and TILs.

## Methods

In a pilot study, women with ESBC were treated with cryo 7-10d before mastectomy (6 pts), single-dose Ipi (10 mg/kg) 8-15d before mastectomy (6 pts), or cryo+Ipi (6 pts). Peripheral blood mononuclear cells (PBMCs) and tumor tissue were obtained pre-mastectomy (immediately preceding cryo and/or 1-5d after Ipi), and at mastectomy. T cell repertoire analysis was conducted on extracted DNA using an Illumina^®^ DNA deep sequencing platform and ImmunoSEQ™ software. Clones comprising ≥0.01% of sample DNA were analyzed, and results are reported descriptively.

## Results

Cryo with or without Ipi was associated with decreases in absolute TIL count (median change: Ipi +6%, cryo -73%, cryo+Ipi -16%). However, cryo+Ipi was associated with the greatest expansion of TIL clones across the range of 10^2^-10^4 ^amplicons (table 1), although no difference was observed by group in PBMC clones. Across all samples, a median of 523 TIL clones increased by ≥10^2 ^amplicons, and a median of 4 TIL clones increased by ≥10^3 ^amplicons. The Ipi/cryo group exceeded the median in 80% (4/5) of cases. 21% of all TIL clones were detectable in time-matched PBMC, whereas 16% of expanding (≥10^2^) TIL clones were detectable in time-matched PBMC.

**Table 1:** Therapy-associated T cell clonal expansion in TILs and PBMCs.

	Median # TIL clones expanding by			Median # PBMC clones expanding by		
	≥10^2 ^amplicons	≥10^3 ^amplicons	≥10^4 ^amplicons	≥10^2 ^amplicons	≥10^3 ^amplicons	≥10^4 ^amplicons
**Cryo**	199	2	0	697	1	0
**Ipi**	750	22	1	545	5	0
**Cryo+Ipi**	2010	85	1	830	1	0

## Conclusion

Cryo plus Ipi expands more TIL clones than either strategy alone. Therapy-associated clonal expansion may be difficult to detect in PBMCs. These data highlight the potential importance of TIL repertoire analysis for the monitoring of pts treated with cryo and/or Ipi in the preoperative setting. In a follow-up randomized study, we will evaluate whether TIL clonal expansion across the 10^2^-10^4 ^range can be used to predict recurrence-free survival.
